# Environmental Performance of Small-Scale Seawater Reverse Osmosis Plant for Rural Area Water Supply

**DOI:** 10.3390/membranes11010040

**Published:** 2021-01-06

**Authors:** Latifah Abdul Ghani, Nora’aini Ali, Ilyanni Syazira Nazaran, Marlia M. Hanafiah

**Affiliations:** 1 Faculty of Business, Economic and Social Development, Universiti Malaysia Terengganu, Kuala Nerus 21030, Terengganu, Malaysia; ysyazira@gmail.com; 2 Faculty of Ocean Engineering Technology and Informatics, Universiti Malaysia Terengganu, Kuala Nerus 21030, Terengganu, Malaysia; 3 Institute of Tropical Aquaculture and Fisheries, Universiti Malaysia Terengganu, Kuala Nerus 21030, Terengganu, Malaysia; 4 Department of Earth Sciences and Environment, Universiti Kebangsaan Malaysia, Bangi 43600, Selangor, Malaysia; mhmarlia@ukm.edu.my; 5 Centre for Tropical Climate Change System, Institute of Climate Change, Universiti Kebangsaan Malaysia, Bangi 43600, Selangor, Malaysia

**Keywords:** life cycle assessment (LCA), desalination, environmental impact, seawater reverse osmosis plant, water supply

## Abstract

Seawater desalination is an alternative technology to provide safe drinking water and to solve water issues in an area having low water quality and limited drinking water supply. Currently, reverse osmosis (RO) is commonly used in the desalination technology and experiencing significant growth. The aim of this study was to analyze the environmental impacts of the seawater reverse osmosis (SWRO) plant installed in Kampung Pantai Senok, Kelantan, as this plant was the first installed in Malaysia. The software SimaPro 8.5 together with the ReCiPe 2016 database were used as tools to evaluate the life cycle assessment (LCA) of the SWRO plant. The results showed that the impact of global warming (3.90 kg CO_2_ eq/year) was the highest, followed by terrestrial ecotoxicity (1.62 kg 1,4-DCB/year) and fossil resource scarcity (1.29 kg oil eq/year). The impact of global warming was caused by the natural gas used to generate the electricity, mainly during the RO process. Reducing the environmental impact can be effectively achieved by decreasing the electricity usage for the seawater desalination process. As a suggestion, electricity generation can be overcome by using a high-flux membrane with other suitable renewable energy for the plant such as solar and wind energy.

## 1. Introduction

Three-quarters of the planet’s surface is covered with water. It is one of the most abundant sources in the world, with 97.5% of water consisting of salt water from the oceans, and 2.5% of the clean water existing in the atmosphere, ice mountains, and ground water [1]. According to Thompson et al. and Taikan and Rose [2,3], one in three people in the world is affected by water scarcity, and nearly one-fifth of the world’s inhabitants live in areas with water shortage problems. Compounded by the increasing global population, industrial development, and agricultural activity, many countries are facing water scarcity and quality problems and thus are unable to meet the demand of providing clean water [4]. Therefore, the available water supply is less than the public demand, and the problem is expected to worsen due to population growth, urbanization, climate change, and accretion in household and industrial use. It is estimated that the global water needs by the year 2030 will rise from 4500 billion cubic meters (m^3^) to 6900 billion m^3^. This amount of the surface water resources is frankly not sufficient for the future generations [5].

Increasing potable water scarcity due to water quality problems and shortages of water supply to the consumers needs to be solved by finding alternative ways. Seawater can be used as one of the alternative ways to overcome the shortage of freshwater supply, especially in rural and urban areas. The production of freshwater from seawater using a membrane-based process includes reverse osmosis, electrodialysis, membrane distillation, and evaporation [5]. The desalination of seawater is the most commonly used method in countries that suffer from a scarcity of potable water [6,7]. Abdel-aal et al. [8] stated that reverse osmosis (RO) was frequently used for seawater and brackish water desalination, water treatment, and wastewater restoration for the past 30 years due to its preferable and stable production of water and low-cost system as compared to others. Commercially developed RO technology for desalination needs a large amount of electricity to power the shaft to generate pump, and the electricity is produced from non-renewable and fossil fuels pollutant.

According to previous studies, the water demand in Malaysia is growing at a rate of 4% annually and is predicted by 2020 to achieve about 20 billion m^3^ [9]. About 25 river basins have been identified as areas with water scarcity problems, and most of the rivers have already reached their maximum capacity and have been polluted at some stages [10]. The residence of Kelantan is facing unsteady water management due to outmoded water conveyance and deficient water storage capacity. Thus, these problems will restrict the conventional allocation of water to the residence. According to the report from the National Water Resources Survey [11], the abstraction rate for drinking water from the Kelantan River was at 60% or 254.074 million liters per day, while from groundwater was at 40% or 176.342 million liters per day. The Malaysian Government planned for the households in Kelantan to obtain clean water from 420 to 800 million liters daily in 2019. A desalination plant was built in Kampung Pantai Senok, Kelantan to provide clean and fresh potable water to the consumers due to the low quality of the groundwater in the rural area.

The environmental burdens caused by the installation of the desalination plant must be evaluated to provide environmentally friendly technology for the future development. Life cycle assessment (LCA) is a suitable tool to evaluate the environmental impacts, such as the depletion of natural resources and the environmental burden from desalination technologies [12,13]. The environmental impacts of the whole life cycle of the product, the process, and the activity can be quantified using LCA [14,15]. According to ISO 14040 [16], research on LCA has been carried out on the water treatment process, including desalination using RO, wastewater, and membrane industries. The main objective of this study focused on identifying the concepts of life cycle assessment and the main sources of environmental impacts during the operational phase of the seawater desalination plant. This study also evaluated the main sources of environmental impact considering the operational phase of the seawater desalination process.

## 2. RO Membranes in Desalination Technologies and Their Novel Theories

A membrane is a thin, semi-permeable layer located between two different phases of separation. Aspects of membrane classification include morphological forms (groups of asymmetric membranes and symmetrical membranes), existence forms (synthetic membranes and natural membranes), module shapes (flat membranes and tube membranes), and pore measurements (macropores, mesopores, and micropores). The membrane processes for the reverse osmosis (RO) technology using one of the membranes welding in terms of driving force are high pressure thrust, as well as microfiltration (MF), ultrafiltration (UF), and piezodialysis [17]. Membrane performance in RO systems is the top choice by the water-treatment industry leaders due to low-energy consumption; application under normal conditions; ease to be combined (hybridized) with other operations; requiring no optional additives; simple and compact membrane module design specifications; and ease of utilizing in its operation [18,19]. For example, two studies by Zhang [20,21] reported that the use of rotating graphene nanoporous membranes with pores of diameter 2 to 4 nanometers successfully performed almost 100% salt rejection by using the RO system.

**Figure 1:**
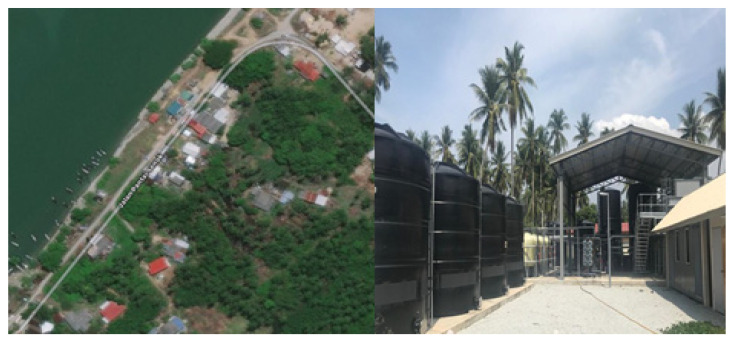
Map of seawater reverse osmosis (SWRO) desalination plant located in Kampung Pantai Senok, Kelantan.

**Figure 2:**
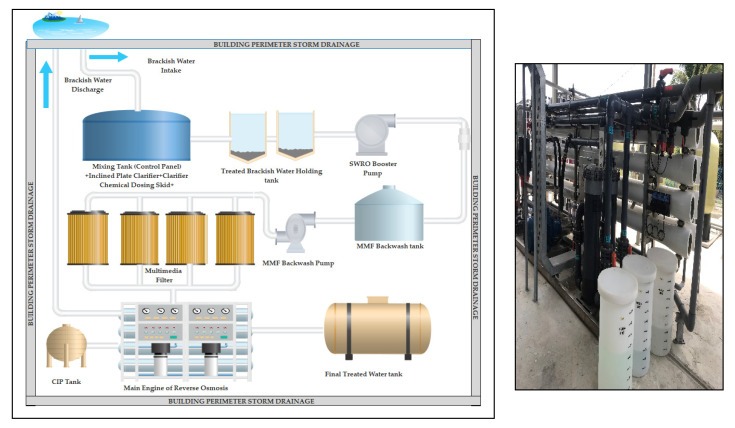
Photograph of reverse osmosis (RO) membrane system (**left**), and design drawings of Senok plant adapted from the original sketch plan of Advanced Membrane Technology Research Centre (AMTEC), Universiti Teknologi Malaysia (UTM) (**right**).

Currently, the membrane RO system is the most well-known technology used in desalination technology. The sea water reverse osmosis (SWRO) equipment is standardized, consisting of membranes, motors, pumps, valves, flow meters, and a pressure gauge. The SWRO system is commonly used, as it only requires a small space due to its modularity, automatic process control, and low cost for water production compared to other systems [20]. Due to the modular design of SWRO systems, the maintenance for the machine can be performed without shutting down the entire plant. SWRO is the process of water passing through a semi-permeable membrane from high salt concentration to a lower salinity solution via osmotic pressure to separate the salt and other dissolved contaminants from water [22]. For SWRO, a high amount of pressure is needed for the seawater osmotic pressure to force water to pass through the membrane [23]. Figure 1 and Figure 2 is a conventional RO plant framework for a desalination technology.

Historically, the development of membrane technology began in 1627 by Sir Francis Bacon. Antonie Van Leewenhoek applied membrane research techniques using a microscope in 1676. Abbe Nollet introduced the semi-permeability concept in 1748. Then, in 1800 and 1804, Fick and Robert Thom [24,25], Sartorius Werke GmbH, Germany popularized small-scale membrane production in the industrial sector in 1950 [26,27]. Loeb and Sorajan created asymmetric membranes in the late 1950s [28], and the membrane was widespread commercialized in the 1960s and 1970s [29,30,31,32,33]. The literature boom also led to the development of decision-making methods designed to manage seawater desalination more efficiently, sustainably, and systematically in the 1990s until now [34,35,36]. Research by Antonio Martin et al. [37] successfully presented an overview of the application of life cycle assessment (LCA) to assess environmental performance and sustainability based on membrane technology processes. In 2005, the study by Raluy et al. [38] successfully used the LCA approach for different commercial desalination technologies, namely Multi-stage flash distillation (MSF), Multiple-effect distillation (MED), and Reverse Osmosis (RO) by modelling the results scores of different material loads, performing control mitigation to the affected variables. The variety of LCA works in the field of membrane desalination and technology has strengthened them as an effective method to assess alternative desalination environments.

To the best of the researchers’ knowledge, this was the first life cycle analysis (LCA) on an SWRO desalination plant in Malaysia that was implemented to lead a sustainable project operating framework, especially in rural areas affected by water supply crisis. Uniquely, LCA assessment using macro and meso approaches in this framework will delve into the evaluation process from “gate to gate”, including five levels of the SWRO system: water intake/water pumping, pre-treatment, reverse osmosis membrane separation, post-treatment, and water distribution. The author expects that the potential implementation of micro-approach to LCA for the performance of the RO hybrid membrane system is able to apply all the information generated from the findings of this study (for example, material input inventory system, uncertainty and equation value, model module, equipment, technology, and related infrastructure) to lead to economic and environmental savings. It is hoped that this LCA framework can be used to combine decision-making criteria from different disciplines, such as engineering, economics, the environment, and membrane commercial planning when seawater desalination investments are considered for expansion in other areas.

## 3. Materials and Methods

### 3.1. Area of Study

The survey of the SWRO desalination plant was conducted in Kampung Pantai Senok at Tawang District on the east of Kelantan with a latitude of 6.168325 and longitude of 102.3452891 [39]. Kampung Pantai Senok (Senok Beach village) had a population of 7680 people living along the coastal area near Pengkalan Datu River, as depicted in Figure 1. The residents of this area were 98% Malay, while the rest were Chinese or not a Malaysian citizen. The main employment sectors of the population were farmers and fishermen. This desalination plant development project was fully funded by the Ministry of Higher Education through the Translation Research Grant Scheme (TRGS) under the Ministry’s Sustainable Water Resources Strategic Research Action Plan. This desalination plant was the first plant installed in Malaysia as an initiative to provide clean and fresh potable water supply for the residents due to the low groundwater quality in the rural area and limited access to clean water. The total area of the SWRO plant installed in Kampung Pantai Senok was 762 square meters (m^2^) in Lot 1968 and 78 m^2^. This plant was able to benefit around 3000 users in the village. With the ability to produce 500,000 L of treated water per day, the water demand of as many as 10,000 people can be accommodated every day.

### 3.2. The Reverse Osmosis (RO) System

Figure 2 describes the existence of the SWRO membrane system within the confines of the LCA study system. The area of the desalination plant was estimated at around 315 m^2^, which was 21 m × 15 m. The SWRO desalination plant had been operating for one year with a capacity for seawater treatment of 0.5 million liters per day or 20.8 cubic meters per hour (m^3^/h) with an expenditure cost of Ringgit Malaysia (RM) 3,200,000. Details of capital costs for the six related phases respectively are as follows: (1) Pumping seawater; RM384,000, (2) Pre-treatment; RM416,000, (3) Desalination; RM1,152,000, (4) Wastewater; RM256,000, (5) Post-treatment; RM 96,000, and (6) Storing and delivering water; RM160,000. At the initial stage, seawater intake was pumped from the Pengkalan Datu River through 52 m of high-density polyethylene (HDPE) pipes. The second stage involved two pre-treatment processes: pre-treatment A and B. For pre-treatment A, brackish water with feed water around 80 m^3^/h was flowed into the mixing tank, inclined plate clarifier, clarifier chemical dosing skid, and seawater holding tank for coagulation, flocculation, and sedimentation processes. For pre-treatment B, the treated brackish water was pumped out of the seawater holding tank to the multimedia filter (MMF). Stage three was the desalination process. The SWRO machine removed all salts, fine particles, suspended particles and dissolved substances including bacteria from the RO feed water and produced fresh drinking water. The remaining salt water left by the SWRO system was dumped and returned to Sungai Pengkalan Datu. The final stage was the post-treatment process, in which the fresh drinking water was sent to the final water tank treatment for the disinfection of bacteria and pathogens using chlorine.

Basically, there were several stages involving preparation before, during, and in the application of RO membrane technology in this study. At the pre-treatment process stage, multimedia filter backwash, consisting of MMF backwash tank (capacity: 25 m^3^ per unit; power: 240/1/50 Hz) and MMF backwash pump (capacity: 35 m^3^/h; pressure gauges 2.5″ × 7 bar) filtered the total suspended solids (TSS) in the RO feed water so that possible damages to the RO membrane can be reduced. The adsorption process in the MMF system also involved two sizes of sand and a type of activated carbon made from coconut shells. At the current stage (operational) of SWRO membrane filtration, the estimated quantity of water product for 1 set of a seawater RO system used was 20.8 m^3^ per hour with permeate TDS; 210 ppm; and recovery of <5%. The spiral wound membrane module configuration was made from thin-film composite (TFC) for the purification process. This TFC consisted of three sections: a top layer (polyamide with 0.2 μm); a middle layer (120–150 μm polyethersulfone or polysulfone porous layer); and a bottom layer (40 μm non-woven fabric support sheet) [40]. In summary, the adaptation of membrane technology using integrated systems during the operating phase of this facility successfully achieved a minimal concentration of concentrated seawater and sludge, which is 614 L per year for concentrated saltwater and 0.5 tonnes per year for sludge.

### 3.3. LCA Method for Seawater Desalination

The environmental burden caused by the desalination plant was determined using the LCA approach. The environmental impacts of desalinated water including the electricity usage, materials, and operation of the desalination plant were calculated. According to ISO 14040 guidelines, LCA was divided into four phases:

(a) Phase 1—Goal and Scope Definition: The goal for this research was to study the hotspot of environmental burdens for the SWRO desalination plant in Kampung Pantai Senok. The LCA approach was used as an evaluation method to analyze the environmental effects for both the installation and operational stages of the plant. The system boundary used in this research was gate to gate, which included the type of chemical and the electricity usage during the operational phase of the plant. The functional unit for this research was 1 m^3^ of desalinated seawater.
(b) Phase 2—Life Cycle Inventory (LCI): This study only involved the operational phase of the seawater desalination process and did not include their piping system, water storage tank, and machinery due to their life time. Referring to Table 1, the LCI analysis contained inputs of chemicals and amount of electricity needed for 1 m^3^ of desalinated water. The data for chemicals used during the operational stage were obtained from Tarnacki et al. [41]. The energy consumption of seawater desalination plant for the operational phase was considered to be 3.1 kWh.
(c) Phase 3—Life Cycle Impact Assessment (LCIA): The life cycle impact assessment was generated using ReCiPe 2016 from the LCA software, SimaPro 8.5. The input data gathered from the inventory were calculated using the software to evaluate the environmental impacts by the plant processes. The results from LCIA would determine the environmental burdens produced during the operational phase. The results were the midpoint impacts, which included 18 categories. The LCA library contained a database of energy consumption, emission, and material data to produce one unit of product.
(d) Phase 4—Interpretation: The last phase of LCA was the interpretation of the results. This step involved the evaluation of the results from the inventory analysis and environmental impact assessment of the life stage process. The final stage of the desalination process was negligible due to the lower environmental load compared to the construction and operational stages of the desalination system. In conclusion, the outcomes and the recommendations for the product or the process were made for future studies and development.

## 4. Results and Discussion

### 4.1. Impact Assessment at the Midpoint Level

In this section, the method midpoint (H) for the Life Cycle Impact Assessment (ReCiPe) was used to assess the environmental impacts comprising of 18 impact categories for the SWRO operational phase, as shown in Table 2. The environmental impact assessment was evaluated and summarized based on the characterization of the main impact categories: source, ecotoxicity, and global warming. Referring to Table 2, climate change, fossil depletion, and human toxicity contributed the highest load of 98% compared to the other impact categories for the entire SWRO operational phase. This result showed that electricity and chemicals were among the main factors that yielded a significant load in the SWRO process. However, the release of halogenic anthropogenic can be classified as low risk at 2.6 × 10−0 kg CO_2_ eq per m^3^ per year, which is 23 times lower than the results reported in De Schryver et al. [42]. According to Huijbregts et al. [43], the ReCiPe output analysis was evaluated based on the hierarchical perspective using a long-term perspective, and the risk of this impact could be minimized by practicing the best management on the systems with identified hotspots.

**Figure 3:**
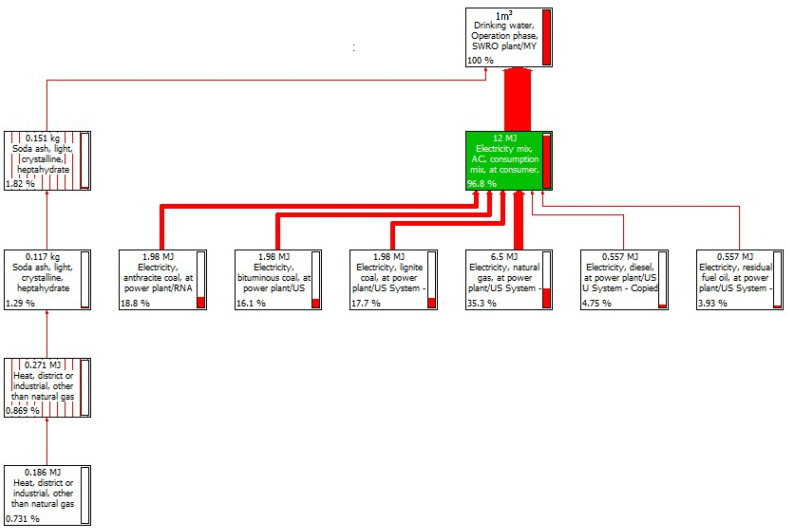
Tree diagram for energy and chemical distribution in SWRO plant.

Figure 3 shows the assessment for the relative energy contribution flow, the chemicals, and the use of membrane on the process impact in the SWRO operational phase. The electricity consumption during the operation of water desalination yielded the highest environmental load at 96%, followed by the use of chemicals and coagulants at 4%. The use of fossil fuels to generate electricity impacted all stages of the SWRO life cycle. The need to redesign the materials such as stainless steel and the use of an integrated electric grid could reduce the environmental impact category, especially on the issue of ozone depletion. Meanwhile, the potential contribution of chemicals and membrane was highest from components including soda ash, sodium hydrogen sulfite, nylon 6-6, glass-filled, sodium hydroxide, and polypropylene resin. According to Hancock et al. [44], the production of a membrane module for a desalination system requires a certain amount of raw materials, including chemicals such as cleaning and antiscalant agents, namely sodium hydroxide (NaOH), hydrochloric acid (HCl), sodium hypochlorite (NaOCl), and sodium tripolyphosphate (Na_5_P_3_O_10_). However, their relative contribution is regularly recorded as low environmental impact. In this study, the contribution of the chemicals was very low compared to that reported in other international studies [45,46]. It should be recognized that the lack of research collaboration between the local LCA community, academic analysts, and industry players will delay the recovery of sources and the control of environmental implications related to the Malaysian desalination sector. Based on this study, further research studies should be conducted on the SWRO management to avoid any additional environmental issues in the future, depending on the qualitative and quantitative assessments, measurement of short and long-term potential exposure, and analysis of more specific options.

### 4.2. Comparison of the Operational Phase Impacts

**Figure 4:**
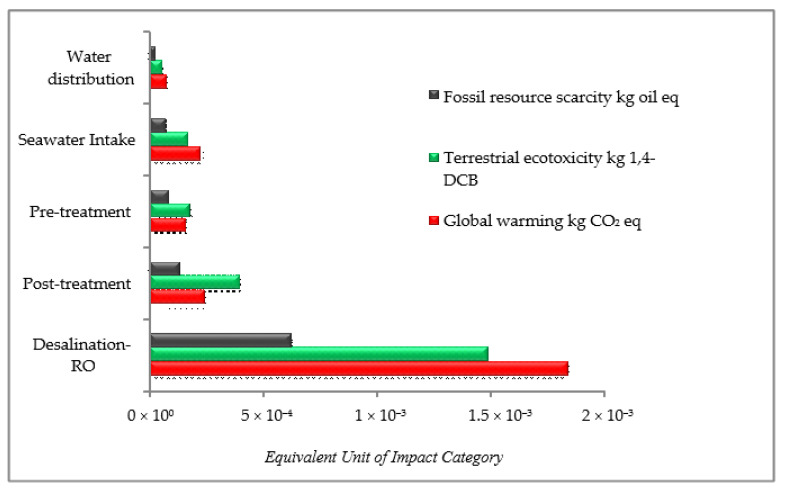
Impact assessment results for the operational phase.

Figure 4 shows the comparison of the relative contribution of impact categories based on the five stages of the Senok SWRO operation. Based on the plot, the terrestrial ecotoxicity and global warming category of the desalination–reverse osmosis and post-treatment stages produced the highest relative contribution to the impact categories in the SWRO process. The total electricity consumption for the membrane and pre-treatment operation for the desalination process was 2.82 kWh/m^3^ with the capacity of around 21 m^3^/h. These results showed that its main impacts were fossil resource scarcity, terrestrial ecotoxicity, and global warming. The results of this study are similar to those reported by other research groups [47,48,49]. According to Sabine and Thomas [50], the electric pump system will experience a large usage increment to force two streams of highly concentrated seawater and brine to freshwater through a permeable membrane. Then, the feed water goes through phases of catchment, removal, and demineralization.

Based on the analysis of the life cycle on the point of risks in the SWRO system, several important summary and recommendations are shown in Table 3.

### 4.3. Explantion of Reverse Osmosis (RO)—Water Quality Effects

Table 4 presents the comparison results of seawater quality analysis before and after treatment using the SWRO membrane technology conducted by AMTEC, Universiti Teknologi Malaysia (UTM). Among some important parameters, such as color, turbidity, boron, hardness, magnesium, sodium and sulfate content, and total dissolved solids, each showed a very different change, with low and good quality content values. Therefore, the removal of weak ions such as boron has met the maximum concentration of boron contained in mineral water of 0.5 mg/L according to the World Health Organization (WHO) standards [51]. According to Alkhudhiri et al. [52], the efficiency and effectiveness of the current SWRO membrane is able to eliminate around 94–96% of boron through the rejection of TDS and water production. Therefore, the application of hybrid SWRO membrane technology such as membrane distillation (MD) at high pH as well as boron adsorbent resin in the second and third stage can save production costs of about 20% in the SWRO membrane systems. This study also showed high trans-membrane pressure (TMP) with the first-pass RO permeate water at a capacity of 28 m^3^/h (pH: 5.7 at boost pressure of 43.7 bar) and second-pass RO permeate water at a capacity of 10 m^3^/h (pH: 5.7 at a power of 37 kW or 415/3/50 Hz). Consequently, this contributed to the increase in the passage of water flux across the membrane as well as the diffusion of better water quality. The RO membrane seems to provide the best results for water purification, reduction of employment, economic savings, and water products that taste more delicious and refreshing. Thus, this LCA case study highlights the importance to fill in key data gaps to further research on the development of the overall LCA approach on the SWRO-UF membrane, specifically at the Senok desalination plant.

### 4.4. Uncertainty Analysis

**Figure 5:**
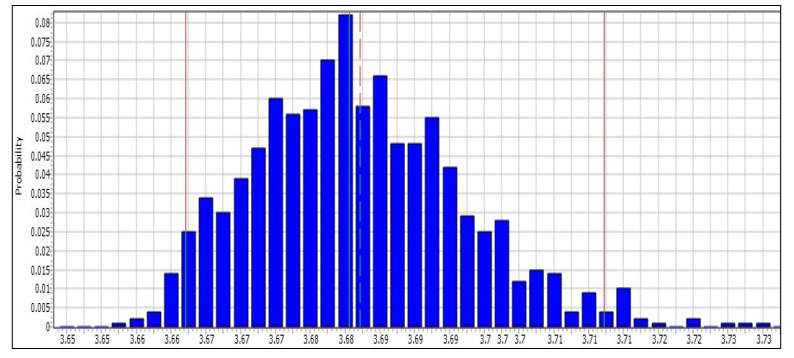
The uncertainty analysis of 1 m^3^ of drinking water from the operational phase in the SWRO plant.

Referring to Figure 5, the Monte Carlo simulation results for the uncertainty analyses by using the SimaPro software algorithm adopted confidence intervals of 95% for 1000 iterations. The results obtained presented the characterization modeling of climate change impact with a mean value of 3.68 kg CO_2_-eq, followed by fossil depletion with 1.28 kg oil eq. This is because these two categories have significant impacts and the highest control over the interpretation of SWRO system analysis. Undeniably, the contributing factors indicated that other categories of impacts such as acidification, N_2_O emission, ecotoxicity, eutrophication, ionizing radiation, ozone and water depletion could cause impact but in a less visible contribution. The LCA results associated with these climate change indicators and fossil depletion can be significantly reduced if a number of contributing factors such as electricity usage during the operational phase are investigated in detail. At 2.5% deviation, the sensitivity value for climate change was 3.66 kg CO_2_-eq, producing cumulative effects of uncertainty that could be improved in terms of the suitability of data profiles, characterization, linear or non-linear modeling, process selection, and others. According to Finnveden [53], uncertainties occur due to selection errors, data inaccuracies, models, and epistemology. For this study, the most significant examples, the electrical profiles and raw input of chemical substance relied heavily on the Ecoinvent database from the European and international research literature. The hypothesis based on the findings of this uncertainty analysis is indeed helpful for the environmental decision-makers to enhance the resilience and sustainability of the desalination plant for a continuous period of time.

## 5. Conclusions

This paper examines whether the difference of load traces inherent in the SWRO system are capable of causing problems and concerns toward the social, economic, and costs aspects of local environment. Currently, there is a shortage in the effort of compiling complete inventory related to the management of input and output of desalination water in Malaysia. Thus, the effects of materials for the operational phase were successfully identified and described as three “hotspots” monitoring, which are electricity based on the use of fossil fuel sources that have a significant impact on input inventory and impact assessment results from the system stages of reverse osmosis, post-treatment, and seawater intake. Utilizing mitigation principles such as improved technology efficiency and the use of renewable source integration can minimize the release of pollutants laden to the environment. Henceforth, applying the principles of choosing the best assessment of emission control based on cost factors and adapting local regulations enforcement for different hybrid SWRO membrane system scales can provide good incentives and reputation to the practitioners of the desalination industry in Malaysia. The vision to provide environmentally friendly and sustainable SWRO technology also requires LCA practitioners to “dissect” and “extract” the justification of other load traces such as carbon footprints, water footprints, and energy footprint in accordance with more accurate literature and estimates. Finally, the author’s main recommendation is to conduct further research specifically on the LCA on SWRO membranes, as this paper has attempted to present the relationship of hot spots in the plant cycle chain with RO membrane existence.

## Figures and Tables

**Table 1:** Inventory of operational phase of SWRO desalination.

Input	Unit	Amount
Input from Nature:		
Seawater	m^3^	1
Inputs from Technosphere:		
Electricity	kWh	3.1
Chlorine	kg	0.001
Hydrochloric acid	kg	0.05
Polyacrymide	kg	0.0024
Polyaluminium chloride	kg	0.0036
Soda ash	kg	0.36
Sodium hydrogen sulfite	kg	0.012
Sodium hydroxide	kg	0.006
Sodium phosphate	kg	0.006

**Table 2:** Overall impact assessment results for operational phase.

Impact Category	Total	Unit
Climate change	2.6 × 10^−0^	kg CO_2_ eq
Ozone depletion	1.0 × 10^−8^	kg CFC^−11^ eq
Terrestrial acidification	3.2 × 10^−2^	kg SO_2_ eq
Freshwater eutrophication	1.2 × 10^−5^	kg P eq
Marine eutrophication	2.7 × 10^−4^	kg N eq
Human toxicity	4.1 × 10^−1^	kg 1,4-DB eq
Photochemical oxidant formation	7.7 × 10^−3^	kg NMVOC
Particulate matter formation	7.1 × 10^−3^	kg PM_10_ eq
Terrestrial ecotoxicity	4.1 × 10^−5^	kg 1,4-DB eq
Freshwater ecotoxicity	4.0 × 10^−3^	kg 1,4-DB eq
Marine ecotoxicity	4.1 × 10^−3^	kg 1,4-DB eq
Ionizing radiation	5.9 × 10^−3^	kBq U_235_ eq
Agricultural land occupation	1.4 × 10^−3^	m^2^a
Urban land occupation	3.3 × 10^−4^	m^2^a
Natural land transformation	6.3 × 10^−6^	m^2^
Water depletion	8.6 × 10^−4^	m^3^
Metal depletion	2.8 × 10^−3^	kg Fe eq
Fossil depletion	9.1 × 10^−1^	kg oil eq

**Table 3:** The alternative options for the mitigation aspects of the SWRO system.

Aspects	Desalination Process	Recommendations/Comments
Energy use	The use of 2 kWh per m^3^ of energy from the Tenaga Nasional Berhad (TNB) grid has the second largest ecological impact especially on the terrestrial ecotoxicity and photochemical oxidation.	○ SWRO plants, on the contrary, only require about 3–4 kWh/m^3^ of electrical energy, and hence, they have significantly lower overall energy demand than distillation plants. ○ Best Available Techniques (BAT) in SWRO plants to minimize energy demand include pressure exchangers and various frequency pumps, besides optimizing the process as a whole. ○ Then, the suggestion of using renewable resources such as wind power from small turbines may have a significant impact on the fresh water and marine aquatic toxicity and human toxicity in the future.
Water use	For 1 m^3^ of water product, the SWRO plant treats 3 m^3^ of feed water with antiscalant (i.e., the entire flow).	○ The SWRO process is characterized by lower consumption of water source per 1 m^3^ of water product and consequently a lower volume of concentrated discharge released into the sea than distillation processes, which have larger cooling water requirements.
Material usage	The impact triggered by the seawater inhalation process using a pump motor on a long pipeline at the Senok SWRO plant shows the value of the most important damage effects on abiotic and eutrophic aquatic ecosystems, ozone depletion, and photochemical oxidation.	○ The material usage and brine disposal have little influence on the overall environmental burden compared to the plant operation in Senok due to the high energy demand of all desalination processes. ○ The disadvantage of the copper–nickel alloys frequently used in the distillation plants is their liability to corrosion, which may result in increased metal discharge into the South China Sea.
Disposal of the concentrate	The concentrated discharge of salt water has the greatest potential for environmental impact in the study area. This is due to the amount of pollutants present in salt solutions containing chemical concentrations and salinity, dissolved oxygen in water, quantity of organic matter, acids, pH, temperature, and effluent, which must be monitored together. The three environmental effects that are considered important in this study area are toxicity to humans and aquatic species, resource extraction, and acidification.	○ It is necessary to distinguish between the salt and chemical additives. The key to avoid impacts of salinity is to sufficiently dilute and disperse the salinity load to ambient concentrations. ○ Mixing and dispersal of the salinity load can be enhanced by installing a multi-port user system in the SWRO plant. ○ Project proponents should develop more specific salt emission regulations pursuant to the legal protocol of the Malaysian Maritime Management Framework. Developers may also require consistent and ad hoc reporting of emission limits, EIA studies, and water mixing zone requirements. This is due to the fact that variables such as distance, area, method of removal of concentrated salt, and effluent should also be considered, as freshwater organisms are toxic to the salty environment. Other studies dealing with concentrations in the body of water, such as membrane-operated ion concentration tests, may reduce anxiety problems over a long period of time. ○ Emphasis should be placed on the incremental aspects of technical and environmental costs, including equipment purchase, installation costs, maintenance costs, staff training costs, and environmental costs.
Chemicals	The impact of each chemical used in the operational stage, including the post-treatment and installation stage can be assessed. According to the results of the normalization for the post-treatment stage, sulfuric acid has the highest environmental impact. Moreover, the results are different for the pre-treatment process. The iron chloride used as a coagulant also has the highest impact on the ozone layer depletion and terrestrial ecotoxicity. Meanwhile, the use of lime for the purpose of remineralization has important criteria in the release of greenhouse gases, leading to a reduction in the ozone layer and an increase in ultraviolet radiation into the air. Finally, the sulfuric acid used for pH monitoring and water quality data recorded elevated chemical contributions, leading to the category of destruction of respiratory damage to humans and land acidification effects.	○ Chlorine can be effectively removed by different chemicals, such as sodium bisulfite, as practiced in the SWRO plants. ○ Filtered backwash water should be treated by dewatering and land-deposition where possible, and cleaning solutions should be treated on-site in special treatment facilities or discharged into a sanitary sewer system.

**Table 4:** Water quality analysis before and after treatment for Senok SWRO desalination.

Parameter	Unit	Senok Seawater	Senok SWRO	Permitted Level *
**Physical Standard**				
pH	-	7.6	6.5	6.5–8.5
Colour	TCU	10	<5	15
Turbidity	NTU	2.7	0.3	2
**Chemical Standard**				
Aluminum (Al)	mg/L	0.05	ND (<0.02)	0.2
Barium (Ba)	mg/L	0.08	ND (<0.02)	0.7
Biocides (Total)	mg/L	ND	ND	0.1
Boron (B)	mg/L	2.7	0.5	0.5
Cadmium (Cd)	mg/L	ND (<0.002)	ND (<0.002)	0.003
Carbon Chloroform Extract	mg/L	ND	ND	0.5
Chloride	mg/L	14,120	110	250
Floride (F)	mg/L	<0.1	<0.1	0.6
Hardness (CaCO_3_)	mg/L	4300	4	500
Iron (Fe)	mg/L	0.19	ND (<0.02)	0.3
Lead (Pb)	mg/L	ND (<0.01)	ND (<0.01)	0.01
Magnesium (Mg)	mg/L	871	0.8	150
Manganese (Mn)	mg/L	0.05	ND (<0.02)	0.1
Mercury (Hg)	mg/L	ND (<0.001)	ND (<0.001)	0.001
Mineral Oil	mg/L	ND	ND	0.3
Nickel (Ni)	mg/L	ND (<0.02)	ND (<0.02)	0.02
Nitrite (NO_2_^−^)	mg/L	<0.1	<0.1	50
Nitrate (NO_3_^−^)	mg/L	<0.1	0.1	10
Nitrate (N)	mg/L	<0.1	<0.1	0.002
Phenol (CH_4_H_2_OH)		ND (<0.002)	ND (<0.002)	0.002
Residual Chlorine (CI_2_)	mg/L	0.13	0.05	≥0.2
Sodium (Na)	mg/L	3793	54	200
Styrene	mg/L	ND	ND	0.2
Sulfate	mg/L	180	4	250
Zinc (Zn)	mg/L	0.06	0.15	3
Total Dissolved Solids	mg/L	17,100	150	-

* The 25th A Schedule of the Food Act 1983 [Subregulation 394 (1)], Food regulations 1985.

## Data Availability

All data generated or analyzed during this study are included in this published article.
